# Factors associated with poor socioeconomic status among Malaysian older adults: an analysis according to urban and rural settings

**DOI:** 10.1186/s12889-019-6866-2

**Published:** 2019-06-13

**Authors:** Suzana Shahar, Divya Vanoh, Arimi Fitri Mat Ludin, Devinder Kaur Ajit Singh, Tengku Aizan Hamid

**Affiliations:** 1 Centre for Healthy Aging and Wellness, Faculty of Health Sciences, Universiti Kebangsaan Malaysia, Jalan Raja Muda Abdul Aziz, 50300 Kuala Lumpur, Malaysia; 2 Nutrition and Dietetics Programme, School of Health Sciences, Health Campus, Universiti Sains Malaysia, 16150 Kubang Kerian, Kelantan Malaysia; 3 Biomedical Science Program, School of Diagnostic and Applied Health Sciences, Faculty of Health Sciences, Universiti Kebangsaan Malaysia, Jalan Raja Muda Abdul Aziz, 50300 Kuala Lumpur, Malaysia; 4 Physiotherapy Programme, School of Rehabilitation Science, Faculty of Health Sciences, Universiti Kebangsaan Malaysia, Jalan Raja Muda Abdul Aziz, 50300 Kuala Lumpur, Malaysia; 5 Malaysian Research Institute on Ageing (MyAgeing), Universiti Putra Malaysia, 43400 Serdang, Selangor Malaysia

**Keywords:** Socioeconomic status, Urban, Rural, Older adults, Disability

## Abstract

**Background:**

Poverty at old age is associated with poor dietary habit, nutritional status and higher rates of chronic diseases and psychosocial problems. However, there is limited information about this matter according to urban and rural settings. The aim of this study was to identify dietary, nutritional, physical and cognitive factors associated with poor socioeconomic status (SES) among older adults according to urban and rural settings in Malaysia.

**Methods:**

An analysis was conducted among 2237 older adults who participated in a longitudinal study on aging (LRGS TUA). This study involved four states in Malaysia, with 49.4% from urban areas. Respondents were divided into three categories of SES based on percentile, stratified according to urban and rural settings. SES was measured using household income.

**Results:**

The prevalence of low SES was higher among older adults in the rural area (50.6%) as compared to the urban area (49.4%). Factors associated with low SES among older adults in an urban setting were low dietary fibre intake (Adj OR:0.91),longer time for the Timed up and Go Test (Adj OR:1.09), greater disability (Adj OR:1.02), less frequent practice of caloric restriction (Adj OR:1.65), lower cognitive processing speed score (Adj OR:0.94) and lower protein intake (Adj OR:0.94). Whilst, among respondents from rural area, the factors associated with low SES were lack of dietary fibre intake (Adj OR:0.79), lower calf circumference (Adj OR: 0.91), lesser fresh fruits intake (Adj OR:0.91), greater disability (Adj OR:1.02) and having lower score in instrumental activities of daily living (Adj OR: 0.92).

**Conclusion:**

Lower SES ismore prevalent in rural areas. Poor dietary intake, lower fitness and disability were common factors associated with low in SES, regardless of settings. Factors associated with low SES identifiedin both the urban and rural areas in our study may be useful inplanning strategies to combat low SES and its related problems among older adults.

## Background

Socioeconomic inequalities have contributed to progressive health problems worldwide [1]. Majority of the older people are retired and have limited income. Their opportunity to work is highly restricted, placing them at a very low levels of socioeconomic status (SES), which may increases their risk of mental health problems especially higher among urban dwellers [2]. Older people with economic disadvantage may have poor cognitive function due to lower educational level [3]. Poor SES is often associated with lower education level. However, as time evolves, changes in parental perception towards children’s education have been observed. Parents with low SES have reported to have equally high expectation towards their children’s education as those in the high SES group [4].

Low SES is often associated with poor nutritional status, mental health problems, disability and even mortality. Economically stable older adults have lower rates of mortality by 15.3 and 10.9% in men and women, respectively and may be due to accessibility to better food and treatment [5, 6]. Study by Doris et al. [7] demonstrated that consumption of healthy diet, regular exercise and proper medical treatment are among the health determinants of older adults.

Aging itself increases risk of malnutrition in older adults due to the simultaneous co-existence of several factors, namely poor oral health, frailty, chronic diseases, physical limitations and psychosocial problems which may gradually deteriorate bodily function [8]. Interference with food availability especially among socioeconomically disadvantaged older adults place them at higher risk of energy and protein deficiencies which may lead to debilitating conditions such as muscle wasting, slower wound healing, anaemia, osteoporosis, and higher risk of hospital admission [9].

Malaysia will be an aged nation by year 2035 and income inequality will become a serious issue among older adults [10]. Older people often categorised as low SES due to unemployment or lack of financial assistance at later life [9]. Earlier studies showed an association between low SES, poor well-being, deteriorating health, lower education level, lack of conducive living environment and limited access to facilities [11]. Survey by Abu Bakar among 1400 older adults around Malaysia showed that poverty is higher in the rural area especially among older women due to lower education level and no proper employment [12].

However, little is known about disparities according to either urban or rural settings. Such information is needed to appropriately plan for programme and resources to alleviate the quality of life of the low income older adults according to settings. Thus, this study aimed to determine the occurrence of low SES according to urban and rural settings and further explorefactorsassociated with low SES from a large scale community based population study.

## Methods

Analysis was conducted on baseline data of the Longitudinal Study on Neuroprotective Model for Healthy Aging among Malaysian Adults (LRGS TUA) involving 2237 community dwelling older adults aged 60 years and above residing in four states in Malaysia. Respondents were chosen using the multi stage random sampling method involving three sampling steps namely the primary sampling unit (PSU), secondary sampling unit (SSU) and tertiary sampling unit (TSU). PSU involves the selection of state, SSU is the selection of census circle within each state, while TSU is the process of selecting living quarters. The detailed methodology of this study has been described earlier [13].

This study involved older people from both the urban and rural areas. Urban area in this study was defined as an area with a total population of at least 10,000 people with at least 60% of population (aged 15 years and above) were not engaged in agricultural activities, while rural area has total population of less than 10,000 people who are mostly involved in agricultural sector [14]. SES was measured using the household income parameter which included pension, money given by spouses, children or others, and welfare assistance. SES was categorised as three groups using the percentile approach. For the purpose of this study, the cut-off points for the three groups were; below MYR 420 (low SES), MYR 420- MYR 1149 (medium SES) and MYR 1150 and above (high SES). Similar cut-offs was applied for older adults from both the urban and rural areas due to the presence of income inequality in both areas.

The inclusion criteria were older adults aged 60 years and above, Malaysian citizen, had no dementia as confirmed by doctors and terminal illnesses and not wheel-chair bound. The exclusion criteria were those with Mini Mental State Examination (MMSE) score 14 and below.

Data that included socio-demography, medical history, nutritional status, cognitive function, fitness, functional status, and psychosocial parameters as summarized in Table 1, was analysed according to SES within urban or rural settings.

**Table 1:** Parameters included in the study

	Parameters
Socio-demography	Name, address, identification card number, gender, ethnicity, education years, living arrangement, marital status, smoking, household income
Medical history	Self-reported chronic diseases such as hypertension, diabetes, hypercholesterolemia, arthritis, heart diseases, asthma, constipation, urinary incontinence, hearing or vision problem
Anthropometry	Body mass index [15], waist circumference, calf circumference
Functional status	Instrumental Activities of Daily Living [16], Activities of Daily Living [17]
Physical Fitness	Timed up and go test, back scratch test, chair stand test, chair sit and reach test, 2 min step test [18]
Dietary intake	Dietary history questionnaire for assessing habitual dietary habits [19]
Cognitive function	Digit span for attention and working memory [20], digit symbol for processing speed [20], MMSE for global function [21], Rey Auditory Verbal Learning Test (RAVLT) for verbal memory [22], Visual Reproduction (VR) for visual memory [23]
Disability	World Health Organization Disability Assessment Schedule 2.0 [24]
Depressive symptoms	Geriatric Depression Scale-15 [25]
Loneliness	Three item loneliness scale [26]

Body Mass Index (BMI) for older adults was categorized as underweight (≤ 23.9 kg/m^2^), normal (24 to 27 kg/m^2^) and overweight (≥27.1 kg/m^2^) [15].

### Statistical analysis

Predictive Analytic Software (PASW) version 22.0 was used for data analysis. Univariate analyses were performed using the cross-tabulation analysis for categorical variables and One Way Between Group ANOVA for the numerical variables. Cross tabulation analysis conducted using Chi-Square test was to determine the association between two categorical variables such as gender and socioeconomic status, while One-Way Between Group ANOVA was to measure the mean differences between the categorical (socioeconomic status) and numerical variables (age, cognitive test scores). Multivariate analysis was conducted using Ordinal Logistic Regression (OLR) with socioeconomic status as the dependent variable. Two OLR models were produced, each representing problems among older adults with low SES in the rural and urban areas respectively. Significance level was set at *p* < 0.05.

## Results

Prevalence of poor SES in the urban area was lower (42.5%) than the rural settings (57.5%) (*p* < 0.001). Respondents who were from the poor SES were older (70.6 ± 6.4), had lower education levels (3.3 ± 3.1), lived alone (16.6%) and were smokers (19.1%) as compared to those in the middle and high SES groups (*p* < 0.05) (Table 2).

**Table 2:** Sociodemographic characteristic based on socioeconomic status [Present as mean ± SD or n(%)]

	Low SES (*n* = 753)	ModerateSES (*n* = 739)	HighSES (*n* = 745)	Total (*N* = 2237)
Age, years (Mean ± SD)	70.5 ± 6.4	68.5 ± 6.1	68.0 ± 5.8	69.0 ± 6.2**
Gender				
Women	454 (60.3)	357 (48.3)	341 (45.8)	1152 (51.5)***
Men	299 (39.7)	382 (51.7)	404 (54.2)	1085 (48.5)
Education years (Mean ± SD)	3.3 ± 3.1	5.1 ± 3.5	7.2 ± 4.3	5.2 ± 4.0***
Residing Location				
Urban	320 (42.5)	366 (49.5)	420 (56.4)	1106 (49.4)***
Rural	433 (57.5)	373 (50.5)	325 (43.6)	1131 (50.6)
Marital Status				
Single	16 (2.1)	13 (1.8)	7 (0.9)	36 (1.6)***
Married	442 (58.7)	531 (71.9)	564 (75.7)	1537 (68.7)
Divorced	19 (2.5)	17 (2.3)	3 (0.4)	39 (1.7)
Widow/Widower	276 (36.7)	178 (24.1)	171 (23.0)	625 (27.9)
Ethnicity				
Malay	478 (63.5)	451 (61.0)	481 (64.6)	1410 (63.0)**
Chinese	253 (33.6)	248 (33.6)	213 (28.6)	714 (31.9)
Indian	21 (2.8)	39 (5.3)	48 (6.4)	108 (4.8)
Others	1 (0.1)	1 (0.1)	3 (0.4)	5 (0.2)
Smoking habit				
Yes	144 (19.1)	137 (18.5)	106 (14.2)	387 (17.3)*
No	609 (80.9)	602 (81.5)	639 (85.8)	1850 (82.7)
Living Arrangement				
Alone	125 (16.6)	72 (9.7)	40 (5.4)	237 (10.6)***
With others	628 (83.4)	667 (90.3)	705 (94.6)	2000 (89.4)

*p < 0.05; **p < 0.01;***p < 0.001

Analysis of the urban respondents demonstrated lower SES among the oldest (70.1 ± 6.1 years old), lowest level of education (3.3 ± 3.4), women (65.3%) and Chinese (65.0%) (*p* < 0.05). Prevalence of asthma was also higher among the low SES (8.8%) respondents as compared to the medium and high SES groups. Besides that, those in the lower SES were nutritionally at risk due to the lowest MUAC (28.1 ± 3.3 cm) and calf circumference (3.31 ± 3.6 cm) (*p* < 0.001). Respondents in the low SES group had lower performance in both cognitive and physical fitness tests (Table 3).

**Table 3:** Sociodemographic characteristic, medical profile, nutritional status, dietary intake and psychosocial profile of urban respondents [Presented as mean ± SD or n(%)]

	Low SES (*n* = 320)	Medium SES (*n* = 366)	High SES (*n* = 420)	Total (*n* = 1106)
Age, years	70.1 ± 6.1	68.5 ± 5.7	67.4 ± 5.7	68.6 ± 5.9***
Education years	3.3 ± 3.4	5.6 ± 3.7	8.3 ± 4.3	6.0 ± 4.4***
Gender				
Women	209 (65.3)	193 (52.7)	198 (47.1)	600 (54.2)***
Men	111 (34.7)	173 (47.3)	222 (52.9)	506 (45.8)
Ethnicity				
Malay	96 (30.0)	129 (35.2)	200 (47.6)	425 (38.4)***
Chinese	208 (65.0)	206 (56.3)	174 (41.4)	588 (53.2)
India & Others	16 (5.0)	31 (8.5)	46 (11.0)	93 (8.4)
Marital status				
Single	11 (3.4)	9 (2.5)	6 (1.4)	26 (2.4)***
Married	204 (63.8)	264 (72.1)	333 (79.3)	801 (72.4)
Divorced	105 (32.8)	93 (25.4)	81 (19.3)	279 (25.2)
Smoking				
Non-smoker	280 (87.5)	308 (84.2)	384 (91.4)	972 (87.9)**
Smoker	40 (12.5)	58 (15.8)	36 (8.6)	134 (12.1)
Living Status				
With others	266 (83.1)	324 (88.5)	401 (95.5)	991 (89.6)***
Alone	54 (16.9)	42 (11.5)	19 (4.5)	115 (10.4)
Medical History				
Diabetes				
No	239 (74.7)	250 (68.3)	310 (73.8)	799 (72.2)
Yes	81 (25.3)	116 (31.7)	110 (26.2)	307 (27.8)
Hypertension				
No	155 (48.4)	171 (46.7)	211 (50.2)	537 (48.6)
Yes	165 (51.6)	195 (53.3)	209 (49.8)	569 (51.4)
Vision or hearing				
No	287 (89.7)	330 (90.2)	389 (92.6)	1006 (91.0)
Yes	33 (10.3)	36 (9.8)	31 (7.4)	100 (9.0)
Urinary incontinence				
No	298 (93.1)	342 (93.4)	386 (91.9)	1026 (92.8)
Yes	22 (6.9)	24 (6.6)	34 (8.1)	80 (7.2)
Constipation				
No	308 (96.3)	348 (95.1)	404 (96.2)	1060 (95.8)
Yes	12 (3.7)	18 (4.9)	16 (3.8)	46 (4.2)
Asthma				
No	292 (91.3)	350 (95.6)	400 (95.2)	1042 (94.2)*
Yes	28 (8.8)	16 (4.4)	20 (4.8)	64 (5.8)
Heart disease				
No	289 (90.3)	327 (89.3)	373 (88.8)	989 (89.4)
Yes	31 (9.7)	39 (10.7)	47 (11.2)	117 (10.6)
Arthritis				
No	231 (72.2)	283 (77.3)	333 (79.3)	847 (76.6)
Yes	89 (27.8)	83 (22.7)	87 (20.7)	259 (23.4)
Stroke				
No	316 (29.0)	359 (98.1)	413 (98.3)	1088 (98.4)
Yes	4 (1.3)	7 (1.9)	7 (1.7)	18 (1.6)
Hypercholesterolemia				
No	220 (68.8)	219 (59.8)	265 (63.1)	704 (63.7)
Yes	100 (31.2)	147 (40.2)	155 (36.9)	402 (36.3)
Anthropometry				
Body Mass Index, kg/m^2^	24.9 ± 4.3	25.0 ± 4.4	25.6 ± 4.4	25.2 ± 4.4
BMI category				
Underweight	142 (45.7)	151 (41.8)	157 (38.0)	450 (41.5)*
Normal	67 (21.5)	115 (31.9)	119 (28.8)	301 (27.7)
Overweight	102 (32.8)	95 (26.3)	137 (33.2)	334 (30.8)
Waist Hip Ratio	0.9 ± 0.1	0.9 ± 0.1	0.9 ± 0.1	0.9 ± 0.1
Weight, kg	59.7 ± 11.9	62.1 ± 11.7	64.7 ± 12.5	62.4 ± 12.3***
Height, cm	154.6 ± 8.1	157.6 ± 8.1	158.8 ± 8.8	157.2 ± 8.5***
MUAC, cm	28.1 ± 3.3	28.7 ± 3.4	29.3 ± 3.6	28.7 ± 3.5***
Waist Circumference, cm	88.1 ± 11.1	88.4 ± 10.7	90.1 ± 11.1	89.0 ± 11.0*
Hip Circumference, cm	96.6 ± 9.1	97.5 ± 9.3	99.3 ± 9.0	97.9 ± 9.2***
Calf Circumference, cm	33.1 ± 3.6	34.0 ± 3.5	35.0 ± 3.8	34.1 ± 3.8***
Cognitive				
Digit span	7.5 ± 2.6	8.0 ± 2.6	8.4 ± 2.5	8.0 ± 2.6***
Best learning RAVLT	36.3 ± 10.5	38.1 ± 10.6	42.4 ± 10.8	39.2 ± 10.9***
Digit symbol	4.5 ± 2.2	5.3 ± 2.6	7.0 ± 3.0	5.7 ± 2.8***
MMSE	22.1 ± 5.3	23.6 ± 4.3	25.3 ± 3.7	23.8 ± 4.6***
Immediate visual memory	39.7 ± 30.7	46.5 ± 33.9	60.6 ± 31.5	49.9 ± 33.2***
Delayed visual memory	31.5 ± 33.0	40.2 ± 35.1	55.6 ± 36.3	43.6 ± 36.4***
Dietary Intake				
Protein, per 1000 kcal/day	43.8 ± 8.7	42.3 ± 8.2	41.4 ± 8.2	42.4 ± 8.4**
Carbohydrate,per 1000 kcal/day	132.7 ± 21.2	136.7 ± 19.2	135.7 ± 20.4	135.2 ± 20.3
Fat, per 1000 kcal/day	32.7 ± 8.4	31.6 ± 7.7	32.2 ± 7.4	32.2 ± 7.8
SFA, per 1000 kcal/day	5.1 ± 3.3	5.1 ± 3.1	5.3 ± 3.1	5.1 ± 3.2
Fibre, per 1000 kcal/day	2.5 ± 1.5	2.7 ± 1.8	3.0 ± 1.9	2.8 ± 1.8
Sugar, per 1000 kcal/day	11.3 ± 8.8	13.5 ± 9.9	16.3 ± 11.4	14.0 ± 10.4
Vitamin C, per 1000 kcal/day	76.2 ± 47.6	82.2 ± 59.6	76.2 ± 50.6	78.2 ± 53.0
Vitamin E, per 1000 kcal/day	6.1 ± 26.9	7.4 ± 32.6	3.3 ± 3.5	5.5 ± 23.8
Folate, per 1000 kcal/day	66.1 ± 44.6	72.1 ± 59.7	72.9 ± 44.1	70.7 ± 50.0
Sodium, per 100 kcal/day	941.2 ± 711.7	863.5 ± 523	856.9 ± 461.3	883.5 ± 566.8
Potassium, per 1000 kcal/day	915.6 ± 308.3	926.8 ± 317.3	944.6 ± 303.8	930.3 ± 309.6
Calcium, per 1000 kcal/day	315.4 ± 137.8	333.9 ± 182.5	333.2 + 159.2	328.3 ± 161.7
Calorie restriction				
No	238 (76.5)	242 (68.0)	239 (58.4)	719 (66.8)***
Yes	73 (23.5)	114 (32.0)	170 (41.6)	357 (33.2)
Fresh fruits intake, (days/week)	3.5 ± 2.5	4.1 ± 2.5	4.8 ± 2.4	4.2 ± 2.5***
Intake of salad or `ulam` (days/week)	6.0 ± 2.0	5.9 ± 2.1	6.2 ± 1.9	6.0 ± 2.0
Psychosocial				
Disability	5.6 ± 3.1	5.4 ± 2.9	5.2 ± 3.1	5.4 ± 3.0***
Depression	3.2 ± 2.5	2.5 ± 2.3	2.3 ± 2.1	2.6 ± 2.3
Loneliness	3.3 ± 1.1	3.2 ± 0.8	3.3 ± 1.0	3.3 ± 1.0
Physical Fitness				
2 min step test, number	58.4 ± 25.6	62.0 ± 26.8	67.3 ± 25.9	63.0 ± 26.3***
Grip strength, kg	22.0 ± 7.5	23.6 ± 7.6	25.0 ± 8.0	23.7 ± 7.8***
Chair stand test, number	10.1 ± 3.1	10.5 ± 11.2	11.2 ± 3.1	10.6 ± 3.1***
TUG, seconds	11.0 ± 2.9	10.2 ± 2.8	9.3 ± 2.5	10.0 ± 2.8***
Chair sit and reach, cm	4.9 ± 12.8	3.6 ± 11.6	3.4 ± 11.2	3.9 ± 11.8
Back scratch test, cm	15.7 ± 13.1	14.5 ± 12.3	12.7 ± 13.4	14.2 ± 13.0**

*Abbreviation*: *TUG* Timed up and go test, *MUAC* Mid upper arm circumference, *MMSE* Mini Mental State Examination, *RAVLT* Rey Auditory Verbal Learning Test

*p < 0.05; **p < 0.01;***p < 0.001

Similar results were demonstrated among the rural respondents. Respondents from the low SES group were generally older (70.9 ± 6.6 years old), had lower education level (3.3 ± 2.8) and were Malays (88.2%) (*p* < 0.001). Respondents in the low SES group had significantly lower performance in all the cognitive and most of the physical fitness (except for back scratch and chair sit and reach with non-significant findings) tests (*p* < 0.05) (Table 4).

**Table 4:** Sociodemographic characteristic, medical profile, nutritional status, dietary intake and psychosocial profile of rural respondents [Presented as mean ± SD or n(%)]

	Low SES (*n* = 325)	Medium SES (*n* = 373)	High SES (*n* = 433)	Total (*n* = 1131)
Age, years	70.9 ± 6.6	68.5 ± 6.5	68.7 ± 5.9	69.5 ± 6.5***
Education, years	3.3 ± 2.8	4.7 ± 3.2	4.4 ± 3.4	4.4 ± 3.4***
Gender				
Women	245 (56.6)	164 (44.0)	143 (44.0)	552 (48.8)***
Men	188 (43.4)	209 (56.0)	182 (31.4)	579 (51.2)
Ethnicity				
Malay	382 (88.2)	322 (86.3)	281 (28.5)	985 (87.1)
Chinese	45 (10.4)	42 (11.3)	39 (12.0)	126 (11.1)
India & Others	6 (1.4)	9 (2.4)	5 (1.5)	18 (1.6)
Marital status				
Single	11 (3.4)	9 (2.5)	6 (1.4)	26 (2.4)***
Married	204 (63.8)	264 (72.1)	333 (79.3)	801 (72.4)
Divorced	105 (32.8)	93 (25.4)	81 (19.3)	279 (25.2)
Smoking				
Non-smoker	280 (87.5)	308 (84.2)	384 (91.4)	972 (87.9)**
Smoker	40 (12.5)	58 (15.8)	36 (8.6)	134 (12.1)
Living Status				
With others	266 (83.1)	324 (88.5)	401 (95.5)	991 (89.6)***
Alone	54 (16.9)	42 (11.5)	19 (4.5)	115 (10.4)
Medical History				
Diabetes				
No	334 (77.1)	277 (74.3)	245 (75.4)	856 (75.7)
Yes	99 (22.9)	96 (25.7)	80 (24.6)	275 (24.3)
Hypertension				
No	209 (48.3)	192 (51.5)	173 (53.2)	574 (50.8)
Yes	224 (51.7)	181 (48.5)	152 (46.8)	557 (49.2)
Vision or hearing				
No	951 (84.1)	312 (83.6)	295 (90.8)	951 (84.1)***
Yes	180 (15.9)	61 (16.4)	30 (9.2)	180 (15.9)
Urinary incontinence				
No	294 (90.5)	327 (87.7)	374 (86.4)	995 (88.0)
Yes	31 (9.5)	46 (12.3)	59 (13.6)	136 (12.0)
Constipation				
No	368 (85.0)	326 (87.4)	296 (91.1)	990 (87.5)*
Yes	65 (15.0)	47 (12.6)	29 (8.9)	141 (12.5)
Asthma				
No	371 (85.7)	336 (90.1)	303 (93.2)	1042 (94.2)**
Yes	62 (14.3)	37 (9.9)	22 (6.8)	64 (5.8)
Heart disease				
No	392 (90.5)	336 (90.1)	286 (88.8)	1014 (89.7)
Yes	41 (9.5)	37 (9.9)	39 (11.2)	117 (10.3)
Arthritis				
No	249 (76.6)	273 (73.2)	307 (70.9)	829 (73.3)
Yes	76 (23.4)	100 (26.8)	126 (29.1)	302 (26.7)
Stroke				
No	316 (98.2)	363 (97.3)	423 (97.7)	1105 (97.7)
Yes	6 (1.8)	10 (2.7)	10 (2.3)	26 (2.3)
Hypercholesterolemia				
No	232 (71.4)	282 (75.6)	338 (78.1)	852 (75.3)
Yes	93 (28.6)	91 (24.4)	95 (21.9)	279 (24.7)
Anthropometry				
Body Mass Index, kg/m^2^	25.7 ± 4.2	24.7 ± 4.1	24.0 ± 4.7	24.7 ± 4.4***
Waist Hip Ratio	0.9 ± 0.1	0.9 ± 0.1	0.9 ± 0.1	0.9 ± 0.1
Weight, kg	56.0 ± 12.1	60.2 ± 11.5	62.8 ± 12.2	59.3 ± 12.2***
Height, cm	152.6 ± 8.7	155.8 ± 8.2	156.2 ± 8.6	154.7 ± 8.7***
MUAC, cm	27.4 ± 3.6	28.3 ± 3.2	28.9 ± 3.4	28.1 ± 3.5***
Waist circumference, cm	86.1 ± 12.3	87.5 ± 10.8	89.3 ± 10.7	87.5 ± 11.4**
Hip circumference, cm	93.0 ± 9.9	95.0 ± 9.0	97.6 ± 9.2	95.0 ± 9.6***
Calf circumference, cm	31.3 ± 3.8	32.7 ± 3.4	33.7 ± 3.6	32.5 ± 3.8***
BMI category, n(%)				
Underweight	232 (54.1)	162 (44.4)	109 (34.4)	503 (45.3)***
Normal	101 (23.5)	107 (29.3)	95 (30.0)	303 (27.3)
Overweight	96 (22.4)	96 (26.3)	113 (35.6)	305 (27.5)
Dietary				
Protein, per 1000 kcal/day	44.8 ± 8.4	43.7 ± 8.5	43.5 ± 8.8	44.0 ± 8.6
Carbohydrate, per 1000 kcal/day	135.6 ± 20.4	135.9 ± 20.1	134.0 ± 19.6	135.2 ± 20.3
Fat, per 1000 kcal/day	31.1 ± 9.1	31.2 ± 7.6	32.2 ± 7.3	31.4 ± 8.1
SFA, per 1000 kcal/day	4.9 ± 2.8	5.0 ± 2.8	4.8 ± 2.8	4.9 ± 2.8
Fibre, per 1000 kcal/day	1.8 ± 1.1	2.1 ± 1.3	2.5 ± 1.4	2.1 ± 1.3
Sugar, per 1000 kcal/day	11.5 ± 9.0	14.0 ± 10.3	14.4 ± 9.0	13.2 ± 9.6
Vitamin C, per 1000 kcal/day	61.2 ± 44.9	64.8 ± 46.0	67.6 ± 43.5	64.2 ± 44.9
Vitamin E, per 1000 kcal/day	6.1 ± 26.9	7.4 ± 32.6	3.3 ± 3.5	5.0 ± 23.9
Folate, per 1000 kcal/day	54.4 ± 37.8	59.6 ± 45.4	66.1 ± 49.1	59.4 ± 44.0**
Sodium, per 100 kcal/day	860.8 ± 590.1	865.7 ± 553.7	875.0 ± 542.2	866.5 ± 564.3
Potassium, per 1000 kcal/day	905.8 ± 257.5	904.5 ± 259.2	919.2 ± 284.8	909.2 ± 265.9
Calcium, per 1000 kcal/day	315.4 ± 137.8	333.9 ± 182.5	333.2 + 159.2	328.3 ± 161.7
Iron, per 1000 kcal/day	8.1 ± 2.7	8.0 ± 2.4	8.5 ± 2.8	8.2 ± 2.7
Calorie restriction				
No	123 (38.4)	143 (39.1)	179 (41.9)	719 (66.8)
Yes	197 (61.6)	223 (33.4)	170 (41.6)	248 (58.1)
Fresh fruits intake (days/week)	2.8 ± 2.2	3.1 ± 2.3	4.0 ± 2.4	3.2 ± 2.3***
Ulam intake per week (days/week)	5.1 ± 2.3	5.5 ± 2.1	5.7 ± 2.1	5.4 ± 2.2***
Psychosocial				
Disability	10.1 ± 10.3	9.9 ± 11.6	6.6 ± 9.4	8.9 ± 10.6
Loneliness	3.3 ± 0.9	3.3 ± 0.9	3.2 ± 0.7	3.3 ± 0.9
Depression	3.1 ± 2.3	2.5 ± 2.1	2.5 ± 2.0	2.7 ± 2.2***
Cognitive Function				
Digit span	6.5 ± 1.9	7.2 ± 2.0	7.6 ± 2.4	7.0 ± 2.2***
Digit symbol	3.5 ± 1.4	4.1 ± 1.7	4.9 ± 2.3	4.1 ± 1.9***
MMSE	20.0 ± 4.9	22.7 ± 4.5	23.7 ± 4.5	22.0 ± 4.9***
Percentile VRI	29.1 ± 28.7	38.1 ± 31.5	43.4 ± 32.2	36.2 ± 31.3***
Percentile VR II	17.1 ± 23.8	28.3 ± 31.7	35.9 ± 35.0	26.3 ± 31.0***
Physical Fitness				
2 min step test, number	54.0 ± 24.9	58.7 ± 25.8	63.9 ± 24.0	58.4 ± 25.3***
Grip strength, kg	20.7 ± 7.4	23.6 ± 7.7	24.1 ± 8.0	22.6 ± 7.8***
Chair stand test, number	8.6 ± 2.9	9.5 ± 2.9	10.0 ± 3.0	9.3 ± 3.0***
Chair sit and reach, cm	−0.30 ± 12.1	−2.0 ± 10.9	−0.3 ± 10.9	−0.8 ± 11.4
TUG, seconds	12.8 ± 4.0	11.4 ± 3.1	11.4 ± 2.9	12.0 ± 3.5***
Back scratch test, cm	17.0 ± 12.2	15.9 ± 12.6	16.5 ± 12.8	16.5 ± 12.5

*Abbreviation*: *TUG* Timed up and go test, *MUAC* Mid upper arm circumference, *MMSE* Mini Mental State Examination, *RAVLT* Rey Auditory Verbal Learning Test

*p < 0.05; **p < 0.01;***p < 0.001

Among the issues found in the urban respondents in the low SES group were low dietary fibre (Adj OR:0.91; 95% CI: 0.84–0.99) and protein (Adj OR: 0.94; 95% CI: 1.01–10.6) intake, longer time to perform TUG test (Adj OR: 1.09; 95% CI: 1.01–1.17), greater disability (Adj OR: 1.02; 95% CI: 1.01–1.04), slower processing speed (Adj OR:0.94; 95% CI: 0.75–0.87) and less frequent practice of calorie restriction (Adj OR: 1.65; 95% CI: 1.17–2.35) (Table 5).

**Table 5:** Predictors of poor socioeconomic status among urban respondents

	*Estimate*	SE	OR (95%CI)	Sig
Dietary Fibre	−0.092	0.044	0.91 (0.84–0.99)	0.035
Protein Intake	0.034	0.011	0.94 (1.01–1.06)	0.001
Timed Up and Go test	0.082	0.038	1.09 (1.01–1.17)	0.033
WHODAS	0.023	0.009	1.02 (1.01–1.04)	0.008
Processing Speed	−0.210	0.037	0.94 (0.75–0.87)	*p* < 0.001
Sunnah fasting				
No	0.505	0.179	1.65 (1.17–2.35)	0.005
Yes (ref)				

*Abbreviation*: *SE* stand error, *WHODAS:* World Health Organization Disability Assessment Schedule Sunnah Fasting: omit food and beverages from dawn to dusk practiced by Muslims besides Ramadhan fasting

*p < 0.05; **p < 0.01;***p < 0.001

Meanwhile, among the rural respondents, lack of dietary fibre intake (Adj OR 0.79; 95%CI: 0.70–0.90), lower calf circumference (Adj OR: 0.91; 95% CI:0.85–0.98), lack of fruits intake (Adj OR: 0.91; 95% CI: 0.86–0.97), greater disability (Adj OR: 1.02; 95% CI: 1.01–1.03), and lower score in IADL (Adj OR: 0.92; 95% CI: 0.85–0.99) (Table 6).

**Table 6:** Determinants of poor socioeconomic status among rural respondents

	*Estimate*	SE	OR (95%CI)	Sig
Dietary Fibre	−0.235	0.065	0.79 (0.70–0.90)	*p* < 0.001
Calf circumference	−0.089	0.035	0.91 (0.85–0.98)	0.012
Fruits intake	−0.09	0.031	0.91 (0.86–0.97)	0.004
WHODAS	0.016	0.006	1.02 (1.01–1.03)	0.015
IADL	−0.084	0.92	0.92 (0.85–0.99)	0.032

*Abbreviation*: *SE* stand error, *WHODAS* World Health Organization Disability Assessment Schedule; *IADL* Instrumental Activities of Daily Living

*p < 0.05; **p < 0.01;***p < 0.001

## Discussion

### Diet and nutritional status

#### Dietary fibre and low SES

In our study, low socioeconomic status (SES) is associated with lower intake of dietary fibre among older people residing in both urban and rural areas. Low SES attenuated poor nutrition knowledge and purchasing choices of older adults, thus leading to poor dietary pattern with lesser consumption of nutritious food high in fibre especially fresh fruits and vegetables [27–29]. Lower fibre intake is common among senior citizens due to failure of achieving the suggested daily servings of fruits and vegetables [30]. In addition, data from the National Health and Morbidity Survey 2011 in Malaysia, conducted among 2752 older people has reported higher prevalence of Malaysian older people did not meet the World Health Organization (WHO) recommendation for fruits and vegetables intake as compared to other developing and developed nations [31]. Another reason for the reduced intake of dietary fibre among older individuals especially in the rural area, may be due to the beliefsof food taboos such as the cool, hot, sharp and gassy food. Consumption of fruits and vegetables have been associated with chronic diseases such as joint pain, gastrointestinal discomfort, and heart burn [32]. Food high in fibre, which is acceptable and affordable for Malaysian older adults have to be identified and promoted for better dietary habits.

#### Fruits intake and low SES

Furthermore, our study results showed that there is lower fruits intake among those staying in the rural areas. Rural areas have very less retail supermarkets and large grocery stores, thus narrowed the purchasing choices of fruits by older adults. Besides that, fruits are generally more expensive than vegetables and not all rural residents plant fruits at home, thus limiting their intake. Moreover, oral related problems such as gum diseases, tooth decay, dentures, mouth or tongue infection and chewing problems may interfere with fruits intake [33, 34].

#### Protein intake and low SES

Adequate protein intake is essential among older adults for maintaining protein balance, reducing skeletal muscle atrophy and prevent functional decline. This is consistent with the study by Gaspareto et al. [35] showing better protein intake among the higher income older people. In our study, lower protein intake is one of the associated factors of lower SES among older adults in the urban area. Although protein rich food such as fish, milk and yogurt were available in the urban area, its price may be expensive for those in the low SES group. Study has shown that older adults consume less fruits, vegetables, milk, meat, poultry and fish as compared to those in the higher SES. Various factors may contribute to this situation namely lack of transport to purchase food, far distance of the shops, staying alone and loneliness [36]. Besides that, low SES urban senior dwellers may lack of awareness of the importance of protein intake in their daily diet. Lack of dietary protein intake may reduce protein synthesis leading to protein breakdown and muscle wasting [37]. Persistent deprivation of protein may result in sarcopenia characterized by severe muscle atrophy and functional limitation [38]. Older adults has to be encouraged to consume protein for promoting feeling of satiety. Higher protein intake may reduce stimulation in the cortico-limbic brain regions such as insula, hippocampus, parahippocampus, and middle pre-frontal cortex, which regulates cravings, reward, food motivation and executive function. Therefore, greater consumption of protein may promote feeling of fullness and reduce appetite [39].

#### Calorie restriction and low SES

Lack of practice of calorie restriction has also been associated with poor SES among older adults living in the urban areas as compared to older adults residing in the rural areas. This could be due to the reason that urban population older adults were mostly non-Muslims/ Malays, of which calorie restriction such as Muslim Sunnah fasting is not part of their practice. It is desirable to promote fasting as a universal healthy lifestyle towards successful aging. Muslim `Sunnah` fasting has various benefits on physical and mental health. A study involving 1993 community dwelling older adults in Malaysia has demonstrated that practice of calorie restriction was associated with lower risk of Mild Cognitive Impairment (MCI). Another local randomized controlled trial involving older Malay men has shown that 3 weeks of `Sunnah` fasting practiced by the subjects were able to produce improvements in body weight, percentage body fat, body mass index, total cholesterol, low density lipoprotein cholesterol and blood pressure [40, 41].

#### Calf circumference and low SES

Deprived nutritional status as indicated by lower calf circumference is another problem among rural older adults with lower SES. Poor transportation facilities in the rural area is one of the barriers for access to food items, thus increasing dependency on locally available resources for their daily intake to save cost [42]. Progressive decline in muscle mass or lean tissue may lead sarcopenia and further deterioration in physical health [43]. Lower SES has been shown to be a predictor of sarcopenia. Lesser consumption of protein rich food may lead to muscle wasting. Protein deficiency following lack of adequate nutritional intake may activate production of inflammatory cytokines aggravating chronic catabolism, thus decreasing muscle mass [44, 45]. Living alone is another factor influencing dietary intake of older adults [46]. Older Malaysian are facing loneliness as they are living alone. Family institutions are responsible to shower care and love for older adults [47, 48].

#### Disability and low SES

Disability is another affected component among older adults residing in both urban and rural settings. Functionality is defined as the capacity of older people to function well in domains such as physical, mental, social, autonomy, and economic independence. Findings from a cross-sectional study in Brazil demonstrated that there was higher level of disability among older women from the low SES group [49]. Disability especially among those with low SES is closely linked with chronic diseases and this may be associated with lack of accessibility to health care resources [50]. In the present study disability might be associated with poor nutritional and functional status, as has been seen among respondents in rural setting. Whilst, disability among older adults from low SES in urban area might be related to poor mobility and cognitive status, assessed using TUG test and cognitive processing speed respectively.

#### IADL limitation and low SES

There were IADL related limitations among older adults in the low SES group in the rural area in our study. This is consistent with finding of a study in India conducted among 252 older adults residing in the rural villages in the Chittoor district, located in Andhra Pradesh [51]. Rural residence, in addition to poor SES had limited access to medical services, insufficient nutrition, and unhealthy lifestyle that is closely associated with functional limitations. In addition, the China Health and Retirement Longitudinal Study (CHARLS) showed that good economic status was one of the protective factors of functional status [52, 53]. IADL involves complex activities such as money handling, transportation, shopping, using telephone or managing medications. These chores may be taken care of by the care givers of older adults, namely their children. On the other hand, older adults in the urban area may have lesser problems with IADL as they may be still be independent in doing these chores as they are familiar with the environment and have accessibility to the shops.

#### Timed-up-and go and low SES

Taking longer time to perform Timed-up-and go (TUG) test was found to be an indicator of poor SES among older adults residing in the urban area. This may be probably associated with the unfavorable built environment [54] and sedentary lifestyle adopted among older adults residing in the urban area [55]. Study by Hurst et al. (2013) [56], found similar findings as theresults, demonstrating an association between poor performance in TUG tests and low SES. TUG test is an important measure of falls risk, frailty, physical disability, cognitive impairment and all-cause mortality [57, 58].

#### Cognitive function and low SES

Slower processing speed has been linked to poor SES among urban older people in our study. The exact mechanism explaining processing speed and SES is unclear. However, it can be associated with poor social interaction, limited access to health care especially memory clinics, unhealthy lifestyles and lack of involvement in mentally stimulating activities. Poor cognitive function was not associated with low SES among the rural respondents in this study. Migration of rural residents to the urban areas may contribute to this finding. Migrants had higher likelihood of adopting Westernized lifestyle such as dietary pattern high in fat and sugar as well as sedentary lifestyle. These unhealthy lifestyle were risk factors were of poor cognitive function [59].

This study has elucidated the differences in factors associated with SES among urban and rural dwellers. Urban older adults have better SES as compared to those residing in the rural areas. Older adults in the urban area had higher education level, good previous employment which made them eligible for pension, bank savings, and insurance. Most importantly, urban older individuals have better accessibility to health care services which enabled them to seek immediate treatment at an earlier stage of diseases, thus prolonging survival [60]. The strength of this study is that it assessed a wide range of parameters via face-to face interview with stratification of geographical location (urban and rural) through a large scale epidemiological study. While, the limitation of this study is the measurement of SES is based solely on self-reported household income. In the future, a more comprehensive indicator of SES such as Multidimensional Poverty Index (MPI) should be adopted. The identified associated factors of low SES in both the urban and rural areas in our study may be useful to tailor specific and appropriate prevention and intervention strategies among older adults.

## Conclusion

Older people with low SES have poorer nutritional status, dietary habits, cognitive and functional status as compared to the higher and middle income groups. However, the associated factors of low SES group differed slightly in their dietary habits and functional status between those residing in the urban and rural areas. Poor nutrition and functional status especially among rural older individuals place them at a higher risk of health problems due to lesser accessibility to proper health care treatment as compared to the urban residents. Older adults in the rural areas should not be neglected from receiving health related information or advice. There is a need for establishing programme and policies to improve health and nutritional status of older adults, particularly for those from the low income and residing in the rural areas.

## Abbreviations

IADL: Instrumental Activities of Daily Living
SES: Socioeconomic status
TUG: Timed Up and Go Test
WHODAS: World Health Organization Disability Assessment Schedule
